# Exploring the Link Between Sleep Duration and Visual Impairment and Major Eye Diseases: National Health and Nutrition Examination Survey 2005–2008

**DOI:** 10.1155/bmri/1229050

**Published:** 2025-12-28

**Authors:** Jingyue Zhang, Tian Xia, Dongmei Zhang, Xi Yang

**Affiliations:** ^1^ Chongqing Three Gorges Medical College, Chongqing, China, sxyyc.net; ^2^ Department of Ophthalmology, Affiliated People′s Hospital of Chongqing Three Gorges Medical College, Chongqing, China

**Keywords:** National Health and Nutrition Examination Survey (NHANES), ocular disease, sleep duration, visual impairment

## Abstract

**Objective:**

This study is aimed at investigating the association between sleep duration and visual impairment, as well as its relationship with major eye diseases, using data from a large‐scale population‐based survey.

**Methods:**

This cross‐sectional study investigated the prevalence of visual impairment and major eye conditions among 5231 individuals aged 40 and older, utilizing data from the NHANES 2005–2008 survey. Sleep duration was categorized as short (< 7 h), normal (7–9 h), and long (> 9 h). Visual impairment was defined as corrected visual acuity < 20/40, and major eye diseases included cataract surgery, diabetic retinopathy, age‐related macular degeneration, glaucoma, any retinopathy, any ocular disease, and any objectively determined ocular disease. Univariate and multivariate logistic regression were used to assess associations between sleep duration, visual impairment, and major eye diseases. Stratified analyses were further conducted based on diabetes and hypertension status.

**Results:**

The long sleep group demonstrated a significantly higher prevalence of vision impairment, cataract surgery, glaucoma, as well as any ocular disease and any objectively determined ocular disease compared to the normal and short sleep groups (all *p* < 0.05). After adjusting for confounders, long sleep duration was significantly associated with visual impairment (OR: 2.44, 95% CI: 1.09–5.49, *p* = 0.035), glaucoma (OR: 3.38, 95% CI: 1.06–10.8, *p* = 0.042), and any objectively determined ocular disease (OR: 2.24, 95% CI: 1.08–4.65, *p* = 0.035). No significant associations were found between short sleep duration and visual impairment or major eye diseases after controlling for confounders. In the nondiabetic population, long sleep was significantly related to glaucoma, any objectively determined ocular disease, and visual impairment. Among hypertensive patients, long sleep was associated with glaucoma.

**Conclusion:**

Long sleep duration is independently associated with visual impairment, glaucoma, and any objectively determined ocular disease. Longitudinal studies are needed to validate current results and explore causal mechanisms.

## 1. Introduction

Vision loss is a significant global public health concern, imposing substantial social and economic burdens on patients while profoundly diminishing their quality of life. Currently, an estimated 43.3 million people worldwide are blind, and approximately 295 million suffer from moderate or severe vision impairment (VI) [1]. By 2025, these numbers are projected to rise to 64 million individuals with blindness and 474 million with moderate to severe visual impairment. The leading causes of visual impairment and blindness in the United States among individuals over the age of 40 include cataract, glaucoma, age‐related macular degeneration (AMD), and diabetic retinopathy (DR) [2, 3]. Identifying potential risk factors for these conditions is therefore imperative.

Sleep represents a complex neural state that influences virtually all tissues, organs, and physiological systems within the body. Healthy sleep contributes to the maintenance of good physical and mental health [4]. However, suboptimal sleep duration has been implicated in a range of health conditions, including dementia, Type 2 diabetes, obesity, cardiovascular diseases, and an increased risk of all‐cause mortality [5]. In terms of ocular health, emerging evidence suggests that prolonged sleep duration may elevate the risk of DR [6, 7]. Furthermore, insufficient sleep duration has been consistently associated with an increased risk of cataracts [8, 9]. The association between sleep duration and major ocular diseases is progressively being elucidated, yet discrepancies persist in the existing literature [10–13].

In this study, we examined data on sleep duration, VI, and major eye diseases in individuals aged 40 and older in the United States. Participants were part of the National Health and Nutrition Examination Survey (NHANES) conducted from 2005 to 2008, which assessed visual acuity, eye disease history, and retinal images for evaluating major ocular conditions. Using this national cross‐sectional dataset, we explored the distribution of visual impairment and eye diseases across different sleep durations and their interrelationships.

## 2. Materials and Methods

### 2.1. Research Subjects

The NHANES is a continuous, interdisciplinary national survey conducted by the National Center for Health Statistics (NCHS) in the United States. It aims to comprehensively understand the health status of US residents and its relationship with nutrition, diseases, and environmental factors. The survey employs a multistage, probability sampling method to select a representative sample that includes individuals of various ages, races, and socioeconomic statuses. NHANES utilizes diverse data collection methods, such as demographic surveys, face‐to‐face interviews, physical measurements, and laboratory tests, to gather comprehensive and reliable health and nutrition information. The NHANES protocol was reviewed and approved by the research ethics review board of the NCHS. Written informed consent was obtained from all participants.

**Figure 1:**
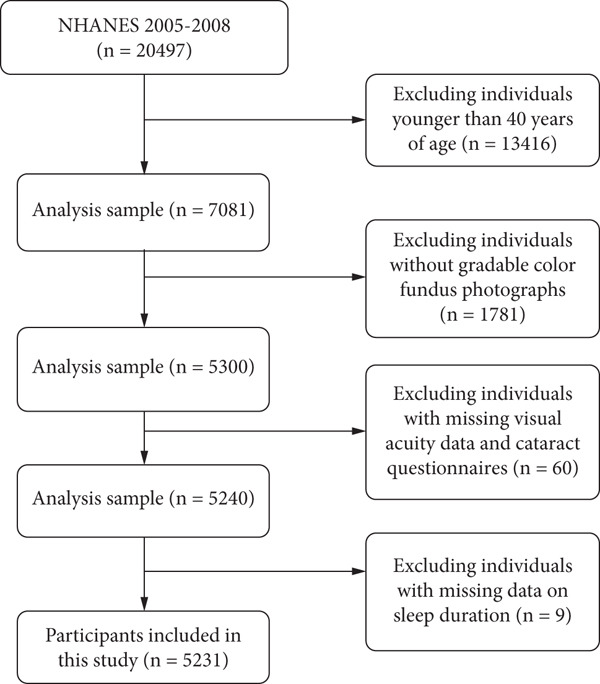
Cohort selection process for the 2005–2008 National Health and Nutrition Examination Survey (NHANES).

We analyzed data from the NHANES study focusing on individuals aged 40 and older to investigate the relationship between sleep duration, major eye diseases, and VI. This study encompasses two NHANES cycles, 2005–2006 and 2007–2008, with a total of 20,497 participants. All included participants had their visual acuity measured, gradable fundus color photographs of the macula and optic disc obtained, and complete sleep duration data collected. We excluded individuals under 40 years of age, those with incomplete demographic information (age, gender, race, education level), and those with missing visual data, cataract questionnaire responses, or sleep duration data. Ultimately, 5231 participants were included for a comprehensive exploration of the relevant associations. The specific screening process is illustrated in Figure 1.

### 2.2. Sleep Duration Assessment

Sleep duration is obtained from the sleep disorders section of the questionnaire, which investigates sleep habits and disorders. The variable label for sleep duration is “How much sleep do you get (hours)?” The inquiry is phrased as “How much sleep {do you/does SP} usually get at night on weekdays or workdays?” The survey uses rounding for sleep duration, with values of 12 h or more being counted as 12 h. Based on previous studies, the short sleep group is defined as having a sleep duration of less than 7 h, normal sleep is defined as 7–9 h, and long sleep is defined as having a sleep duration of more than 9 h [14, 15].

### 2.3. Assessment of Visual Impairment

Visual acuity measurements have been reported in previous studies [16]. The examinees tested participants′ visual acuity with customary corrections, such as eyeglasses, contact lenses, or no correction. This assessment was conducted using an autorefractor (ARK‐760, Nidek Co. Ltd.), which includes built‐in visual acuity charts with lines for 20/20, 20/25, 20/30, 20/40, 20/50, 20/60, 20/80, and 20/200. If a participant′s visual acuity was 20/30 or worse with customary correction, further assessment was performed using objective refractive measurements as the participant′s “correction factor.” Visual impairment was defined as a corrected visual acuity of less than 20/40 in the better eye, corresponding to a presenting distance visual acuity of 20/50 or worse in the better seeing eye [17, 18].

### 2.4. Evaluation of Major Eye Diseases

In the 2005–2008 NHANES survey, respondents aged 40 years and older underwent 45° nonmydriatic retinal imaging of both eyes using a Canon nonmydriatic retinal camera CR6‐45NM. Two images were obtained for each eye: one centered on the macula and the other on the optic disc. Trained technicians captured the images in a dimly lit room, allowing the pupils to dilate naturally. The digital images were then reviewed by graders at the University of Wisconsin and evaluated for DR, AMD, and other retinal diseases.

AMD was diagnosed according to the modified Wisconsin classification scheme [19]. There are three categories: no AMD, early AMD, and late AMD. Early AMD is characterized by the presence or absence of drusen and/or pigmentary abnormalities, while late AMD is defined by the presence of signs of exudative AMD and/or geographic atrophy. We reclassified these diagnoses into two groups: no AMD and the presence of AMD.

The severity of retinopathy is determined by the Early Treatment Diabetic Retinopathy Study (ETDRS) score [20, 21]. A retinopathy level of less than 14 (10–13) is classified as no retinopathy, whereas a score of 14 or higher (14–80) indicates the presence of retinopathy. This score is integrated with the diabetes diagnosis to ascertain whether the patient has DR. Diabetes mellitus is diagnosed if any of the following criteria are met: (a) a physician has confirmed the diagnosis, (b) glycated hemoglobin (HbA1c) level ≥ 6.5%, (c) fasting blood glucose level ≥ 7.0 mmol/L, (d) random blood glucose level ≥ 11.1 mmol/L, (e) 2‐h oral glucose tolerance test blood glucose level ≥ 11.1 mmol/L, or (f) use of antidiabetic medications or insulin. The classifications for DR and AMD are based on the worse eye, defined as the eye exhibiting the more severe retinopathy.

The vertical cup‐to‐disc ratio (CDR) was initially assessed by graders at the University of Wisconsin. In 2012, ophthalmologists at Johns Hopkins University reevaluated images with a CDR of 0.6 or higher, providing additional indicators suggestive of glaucoma, including quality, vertical CDR, glaucoma, disc hemorrhage, size, excavation, notch, and tilt. In this study, a CDR of 0.6 was used to establish the diagnosis of glaucomatous optic neuropathy, with participants exhibiting a CDR of less than 0.6 in both eyes considered free of glaucoma.

Cataract surgeries were self‐reported by survey participants based on their responses to the questions: “Have you ever had a cataract operation?” or “Have you ever had eye surgery to treat cataracts?”

The presence of a history of cataract surgery, any retinopathy, AMD, or glaucoma was categorized as “any ocular disease.” The presence of any retinopathy, AMD, or glaucoma was categorized as “any objectively determined ocular disease.”

### 2.5. Confounding Variable

The potential confounding variables include age, gender, race (non‐Hispanic White, non‐Hispanic Black, Mexican American, and other), education level (less than 9th grade, Grades 9–12, and college or above), poverty status (income at or above the poverty level and other), marital status (married or living with a partner and other), smoking status (current smoker, former smoker, and never smoker), alcohol consumption (nondrinker, 1–5 drinks/month, 5–10 drinks/month, and 10+ drinks/month), body mass index (underweight [< 18.5], normal [18.5–< 25], overweight [25–< 30], and obese [≥ 30]), diabetes status and duration, HbA1c, hypertension, coronary heart disease, and angina. Definitions for some of the confounding variables are available in Table S1.

### 2.6. Statistical Analysis

Given that the NHANES data employs a stratified multistage probability sampling design, we applied weighting during the analysis. The weights used include SDMVPSU (primary sampling unit), SDMVSTRA (stratification), and WTMEC2YR (examination weight). Continuous variables are presented as weighted means ± standard deviations, and differences among the three sleep duration groups were assessed using ANOVA. Categorical variables are reported as sample counts and weighted percentages, with statistical differences evaluated via the chi‐squared test with Rao and Scott′s second‐order correction, and pairwise comparisons between groups were analyzed using Bonferroni′s correction. To further investigate the association between different sleep durations and visual impairments or major eye diseases, logistic regression models were employed to assess the risk of these conditions in short and long sleep groups compared to normal sleep. The crude model did not adjust for any variables and directly analyzed the relationship between sleep duration and the specified conditions. Model 1 adjusted for demographic characteristics, including age, gender, race, education level, marital status, and poverty status. Model 2 further adjusted for lifestyle and health‐related factors such as smoking, alcohol consumption, BMI, diabetes, hypertension, coronary heart disease, and angina based on Model 1. Subgroup analyses were conducted to examine the potential interactions between sleep duration, eye diseases, and the presence or absence of diabetes and hypertension. In the diabetic subgroup, adjustments were made for diabetes duration and HbA1c levels. All statistical analyses were performed using R software (Version 4.0.5, R Foundation, Vienna, Austria, https://www.r-project.org/). A two‐tailed *p* value < 0.05 was considered statistically significant.

## 3. Results

### 3.1. Basic Demographic Information of the Study Participants

In the 2005–2008 NHANES study, there was a total of 7081 participants aged 40 years and older. Due to missing gradable fundus photographs, vision data, cataract questionnaire responses, or sleep duration data, 1850 participants were excluded (Figure 1). Ultimately, 5231 participants were included in the final analysis. The weighted mean age of these participants was 56 ± 11 years, with 2627 males (53%) and 2604 females (47%). The racial distribution was as follows: 77% non‐Hispanic White, 9.6% non‐Hispanic Black, 5.5% Mexican American, and 7.9% other racial groups. Based on sleep duration, 3037 participants (61%) were classified as normal sleepers, 2083 (37%) as short sleepers, and 111 (1.7%) as long sleepers. The characteristics of the study population, categorized by sleep duration, are shown in Table 1.

**Table 1:** Overall demographic and clinical characteristics of included participants and stratified characteristics by sleep duration.

**Characteristic**		**Sleep duration**			**p** **value** ^**b**^
	**Overall,** **N** = 5231 **(100%)** ^**a**^	**Normal sleep,** **N** = 3037 **(61%)** ^**a**^	**Short sleep,** **N** = 2083 **(37%)** ^**a**^	**Long sleep,** **N** = 111 **(1.7%)** ^**a**^	
Age (y), mean ± SD	56 ± 11	56 ± 12	55 ± 11	60 ± 15	0.005
Gender, *n* (%)					0.034
Female	2627 (53%)	1561 (54%)	1006 (50%)	60 (64%)	
Male	2604 (47%)	1476 (46%)	1077 (50%)	51 (36%)	
Race, *n* (%)					< 0.001
Non‐Hispanic White	2801 (77%)	1789 (82%)	948 (69%)	64 (76%)	
Non‐Hispanic Black	1065 (9.6%)	461 (6.4%)	576 (14%)	28 (15%)	
Mexican American	820 (5.5%)	496 (5.3%)	314 (5.8%)	10 (3.8%)	
Other	545 (7.9%)	291 (6.7%)	245 (10%)	9 (5.2%)	
Educational level, *n* (%)					< 0.001
Less than 9th grade	712 (6.5%)	420 (6.4%)	269 (6.4%)	23 (14%)	
Grades 9–12	2090 (37%)	1167 (35%)	874 (41%)	49 (45%)	
College or above	2429 (56%)	1450 (59%)	940 (53%)	39 (40%)	
PIR, mean ± SD	3.33 ± 1.58	3.44 ± 1.54	3.19 ± 1.62	2.47 ± 1.44	< 0.001
PIR status, *n* (%)					< 0.001
Income at or above poverty	4118 (91%)	2440 (93%)	1594 (89%)	84 (85%)	
Other	751 (9.0%)	390 (7.3%)	340 (11%)	21 (15%)	
Marital status, *n* (%)					< 0.001
Married or living with a partner	3369 (70%)	2049 (73%)	1261 (66%)	59 (57%)	
Other	1860 (30%)	987 (27%)	821 (34%)	52 (43%)	
Smoking status, *n* (%)					0.11
1–5 drinks/month	2347 (46%)	1374 (46%)	928 (46%)	45 (41%)	
5–10 drinks/month	339 (8.1%)	187 (8.1%)	147 (8.4%)	5 (2.7%)	
10+ drinks/month	814 (19%)	517 (20%)	284 (17%)	13 (16%)	
Nondrinker	1613 (28%)	894 (26%)	673 (29%)	46 (41%)	
Alcohol consumption, *n* (%)					< 0.001
Current smoker	1058 (21%)	528 (18%)	495 (25%)	35 (32%)	
Former smoker	1682 (31%)	1033 (32%)	619 (29%)	30 (20%)	
Never smoker	2488 (49%)	1475 (50%)	967 (46%)	46 (48%)	
BMI, mean ± SD	29 ± 7	29 ± 6	30 ± 7	29 ± 12	< 0.001
BMI group, *n* (%)					< 0.001
Underweight (< 18.5)	70 (1.3%)	40 (1.4%)	26 (1.0%)	4 (3.2%)	
Normal (18.5–< 25)	1259 (26%)	774 (28%)	449 (23%)	36 (36%)	
Overweight (25–< 30)	1867 (35%)	1092 (35%)	740 (36%)	35 (33%)	
Obese (30 or greater)	1997 (37%)	1110 (36%)	853 (39%)	34 (28%)	
Diabetes mellitus, *n* (%)	1139 (16%)	619 (14%)	490 (18%)	30 (21%)	0.003
Diabetes duration (y), mean ± SD	10 ± 10	10 ± 10	9 ± 9	19 ± 23	0.14
HbA1c (%), mean ± SD	5.66 ± 0.89	5.63 ± 0.86	5.71 ± 0.93	5.59 ± 0.92	0.012
HBP, *n* (%)	3451 (62%)	1952 (61%)	1420 (64%)	79 (65%)	0.2
SBP (mmHg), mean ± SD	126 ± 18	125 ± 18	126 ± 18	130 ± 23	0.11
DBP (mmHg), mean ± SD	72 ± 13	72 ± 12	73 ± 13	70 ± 15	0.070
Coronary heart disease, *n* (%)	294 (4.7%)	169 (4.6%)	115 (4.7%)	10 (8.1%)	0.3
Angina, *n* (%)	208 (3.2%)	114 (2.8%)	89 (3.8%)	5 (5.0%)	0.12
Visual impairment, *n* (%)	98 (1.1%)	53 (0.9%)	38 (1.1%)	7 (4.6%)	< 0.001
Cataract surgery, *n* (%)	632 (9.1%)	391 (9.3%)	215 (8.1%)	26 (20%)	0.006
DR, *n* (%)	346 (4.2%)	176 (3.6%)	158 (4.9%)	12 (8.5%)	0.004
AMD, *n* (%)	394 (6.3%)	234 (6.3%)	145 (6.1%)	15 (13%)	0.10
Glaucoma, *n* (%)	148 (2.0%)	81 (1.9%)	59 (1.8%)	8 (7.2%)	0.007
Any retinopathy, *n* (%)	655 (9.7%)	345 (8.7%)	293 (11%)	17 (15%)	0.009
Any ocular disease, *n* (%)	984 (15%)	593 (15%)	354 (14%)	37 (30%)	0.005
Any objectively determined ocular disease, *n* (%)	524 (8.0%)	303 (7.9%)	198 (7.7%)	23 (20%)	0.005

*Note:* All proportions are weighted estimates of the US population characteristics, taking into account the complex sampling design of the National Health and Nutrition Examination Survey.

Abbreviations: AMD, age‐related macular degeneration; BMI, body mass index; DBP, diastolic blood pressure; DR, diabetic retinopathy; HbA1c, glycated hemoglobin; HBP, high blood pressure; PIR, poverty income ratio; SBP, systolic blood pressure.

^a^Weighted means ± standard deviations for continuous variables; sample counts and weighted percentages for categorical variables.

^b^ANOVA for continuous variables; chi‐squared test with Rao and Scott′s second‐order correction for categorical variables.

### 3.2. Differences Between Sleep Duration Groups

There were no significant differences in the prevalence of hypertension, coronary heart disease, or angina among the normal sleep, short sleep, and long sleep groups. Based on pairwise comparisons using the chi‐square test with Bonferroni′s correction, significant differences in the incidence of ocular diseases were observed across different sleep duration groups. Specifically, the long sleep group was more prone to VI, cataract surgery, glaucoma, as well as any ocular disease and any objectively determined ocular disease, compared to the normal sleep and short sleep groups (all *p* < 0.05). The short sleep group was more likely to develop DR and any retinopathy but less likely to have cataract surgery compared to the normal sleep group (both *p* < 0.05).

### 3.3. Univariate and Multivariate Regression Analyses: The Relationship Between Sleep Duration Groups and Visual Impairment and Eye Diseases

The results of logistic regression models analyzing the relationship between visual impairment, major eye diseases, and various sleep duration groups are detailed in Table 2. In the unadjusted model, compared to the normal sleep group, the short sleep group had a higher likelihood of developing any retinopathy, while the long sleep group showed a higher likelihood of cataract surgery, glaucoma, any objectively determined ocular disease, and visual impairment. In Model 1, adjusted for age, sex, race, education level, marital status, and poverty level, short sleep was significantly associated with any retinopathy (odds ratio [OR]: 1.25, 95% CI: 1.02–1.54, *p* = 0.034) compared to normal sleep, while long sleep was significantly associated with any objectively determined ocular disease (OR: 2.08, 95% CI: 1.10–3.95, *p* = 0.027) and visual impairment (OR: 1.98, 95% CI: 1.05–3.76, *p* = 0.037). However, the associations between long sleep and cataract surgery or glaucoma lost statistical significance. After further adjusting for smoking, alcohol consumption, BMI, diabetes, hypertension, coronary heart disease, and angina, the association between short sleep and any retinopathy was no longer significant. At this stage, the associations between long sleep and any objectively determined ocular disease (OR: 2.24, 95% CI: 1.08–4.65, *p* = 0.035) as well as visual impairment (OR: 2.44, 95% CI: 1.09–5.49, *p* = 0.035) became stronger. Additionally, a significant association was found between long sleep and glaucoma (OR = 3.38, 95% CI: 1.06–10.8, *p* = 0.042).

**Table 2:** Association between visual impairment, major eye diseases, and sleep duration.

	**Crude model** ^**a**^				**Model 1** ^**b**^				**Model 2** ^**c**^			
**Sleep duration**	**Short sleep**		**Long sleep**		**Short sleep**		**Long sleep**		**Short sleep**		**Long sleep**	
**Ocular conditions**	**OR (95% CI)**	**p** **value**	**OR (95% CI)**	**p** **value**	**OR (95% CI)**	**p** **value**	**OR (95% CI)**	**p** **value**	**OR (95% CI)**	**p** **value**	**OR (95% CI)**	**p** **value**
Cataract surgery	0.86 (0.66, 1.11)	0.2	**2.48 (1.29, 4.75)**	**0.008**	1.19 (0.83, 1.71)	0.3	1.19 (0.40, 3.51)	0.7	1.17 (0.78, 1.74)	0.4	1.38 (0.38, 5.02)	0.6
AMD	1.05 (0.96, 1.16)	0.3	1.13 (0.91, 1.41)	0.3	1.01 (0.92, 1.11)	0.8	1.03 (0.82, 1.31)	0.8	1.01 (0.91, 1.11)	0.9	1.03 (0.80, 1.32)	0.8
DR	1.05 (0.95, 1.16)	0.3	1.12 (0.89, 1.42)	0.3	1.01 (0.92, 1.11)	0.9	1.03 (0.79, 1.33)	0.8	1.01 (0.91, 1.11)	> 0.9	1.02 (0.78, 1.35)	0.9
Glaucoma	1.10 (0.65, 1.88)	0.7	**2.63 (1.04, 6.68)**	**0.042**	1.04 (0.59, 1.83)	0.9	2.76 (0.99, 7.65)	0.052	1.01 (0.54, 1.92)	> 0.9	**3.38 (1.06, 10.8)**	**0.042**
Any objectively determined ocular disease	0.97 (0.72, 1.31)	0.8	**2.87 (1.71, 4.81)**	**< 0.001**	1.13 (0.81, 1.56)	0.5	**2.08 (1.10, 3.95)**	**0.027**	1.14 (0.80, 1.61)	0.4	**2.24 (1.08, 4.65)**	**0.035**
Any ocular disease	0.90 (0.72, 1.13)	0.4	2.40 (1.38, 4.17)	0.003	1.14 (0.83, 1.56)	0.4	1.65 (0.62, 4.37)	0.3	1.14 (0.81, 1.62)	0.4	1.65 (0.52, 5.26)	0.3
Vision impairment	1.18 (0.69, 2.00)	0.5	**5.04 (2.80, 9.08)**	**< 0.001**	1.38 (0.78, 2.44)	0.3	**1.98 (1.05, 3.76)**	**0.037**	1.49 (0.77, 2.88)	0.2	**2.44 (1.09, 5.49)**	**0.035**
Any retinopathy	**1.32 (1.10, 1.58)**	**0.004**	1.80 (0.89, 3.64)	0.10	**1.25 (1.02, 1.54)**	**0.034**	1.53 (0.77, 3.04)	0.2	1.18 (0.93, 1.49)	0.2	1.45 (0.68, 3.10)	0.3

*Note:* The ORs are all referenced to the normal sleep duration group. Boldface indicates statistical significance.

Abbreviations: AMD, age‐related macular degeneration; CI, confidence interval; DR, diabetic retinopathy; OR, odds ratio.

^a^Crude Model: not adjusted for any variables.

^b^Model 1: adjusted for age, gender, race, education level, marital status, and poverty status.

^c^Model 2: further adjusted for smoking, alcohol consumption, body mass index, diabetes, hypertension, coronary heart disease, and angina, based on Model 1.

### 3.4. Regression Analysis of Different Subgroups

After adjusting for multiple confounding factors, including age, sex, race, education level, marital status, poverty level, smoking, alcohol consumption, BMI, diabetes, hypertension, coronary heart disease, and angina, we performed stratified analyses based on diabetes and hypertension status (Table 3). Among nondiabetic individuals, long sleep was significantly associated with glaucoma (OR: 4.00, 95% CI: 1.16–13.9, *p* = 0.032), any objectively determined ocular disease (OR: 3.02, 95% CI: 1.27–7.18, *p* = 0.018), and visual impairment (OR: 4.49, 95% CI: 1.63–12.4, *p* = 0.008). However, among individuals with diabetes, no significant associations were found between short or long sleep and visual impairment or major eye diseases. Among individuals with hypertension, long sleep was significantly associated with glaucoma (OR: 4.79, 95% CI: 1.37–16.7, *p* = 0.020). In contrast, among individuals without hypertension, no associations were found between short or long sleep and visual impairment or major eye diseases (Table 3).

**Table 3:** Association between visual impairment, major eye diseases, and sleep duration stratified by diabetes and hypertension status.

	**Diabetes**								**Hypertension**							
	**Present** ^**a**^ **(** **n** = 1154**)**				**Absent (** **n** = 4077**)**				**Present (** **n** = 3451**)**				**Absent (** **n** = 1780**)**			
**Sleep duration**	**Short sleep**		**Long sleep**		**Short sleep**		**Long sleep**		**Short sleep**		**Long sleep**		**Short sleep**		**Long sleep**	
**Ocular conditions**	**OR (95% CI)**	**p** **value**	**OR (95% CI)**	**p** **value**	**OR (95% CI)**	**p** **value**	**OR (95% CI)**	**p** **value**	**OR (95% CI)**	**p** **value**	**OR (95% CI)**	**p** **value**	**OR (95% CI)**	**p** **value**	**OR (95% CI)**	**p** **value**
Cataract surgery	0.90 (0.40, 2.03)	0.8	0.40 (0.01, 16.2)	0.6	1.22 (0.78, 1.93)	0.3	1.61 (0.29, 9.02)	0.5	1.22 (0.75, 1.98)	0.4	1.07 (0.42, 2.77)	0.9	0.92 (0.43, 1.96)	0.8	5.33 (0.16, 180)	0.3
AMD	1.00 (0.78, 1.29)	> 0.9	1.00 (0.52, 1.91)	> 0.9	1.00 (0.92, 1.10)	> 0.9	1.04 (0.83, 1.32)	0.7	1.01 (0.91, 1.12)	0.9	1.03 (0.78, 1.37)	0.8	1.01 (0.88, 1.15)	> 0.9	1.02 (0.68, 1.54)	> 0.9
DR	1.00 (0.78, 1.29)	> 0.9	0.97 (0.47, 2.01)	> 0.9	1.00 (0.91, 1.10)	> 0.9	1.04 (0.83, 1.31)	0.7	1.01 (0.91, 1.12)	> 0.9	1.02 (0.74, 1.40)	0.9	1.01 (0.88, 1.15)	> 0.9	1.02 (0.68, 1.54)	> 0.9
Glaucoma	1.25 (0.22, 7.15)	0.8	2.32 (0.43, 12.7)	0.3	0.91 (0.44, 1.90)	0.8	**4.00 (1.16, 13.9)**	**0.032**	1.22 (0.60, 2.46)	0.5	**4.79 (1.37, 16.7)**	**0.020**	0.69 (0.28, 1.65)	0.4	0.62 (0.06, 7.04)	0.7
Any objectively determined ocular disease	1.67 (0.54, 5.21)	0.3	0.64 (0.09, 4.62)	0.6	1.23 (0.84, 1.81)	0.2	**3.02 (1.27, 7.18)**	**0.018**	1.17 (0.79, 1.75)	0.4	1.92 (0.92, 4.02)	0.075	1.08 (0.65, 1.80)	0.7	4.14 (0.53, 32.2)	0.2
Any ocular disease	1.28 (0.55, 3.01)	0.5	0.25 (0.02, 2.94)	0.2	1.21 (0.84, 1.75)	0.3	2.48 (0.59, 10.4)	0.2	1.17 (0.75, 1.84)	0.4	1.12 (0.47, 2.67)	0.8	1.02 (0.63, 1.64)	> 0.9	6.57 (0.85, 50.8)	0.067
Vision impairment	0.84 (0.26, 2.73)	0.7	0.22 (0.01, 5.71)	0.3	2.01 (0.93, 4.33)	0.070	**4.49 (1.63, 12.4)**	**0.008**	1.52 (0.63, 3.68)	0.3	2.01 (0.67, 6.10)	0.2	1.68 (0.31, 8.98)	0.5	5.98 (0.26, 139)	0.2
Any retinopathy	1.32 (0.81, 2.16)	0.2	1.22 (0.20, 7.30)	0.8	1.20 (0.92, 1.58)	0.2	1.19 (0.28, 5.04)	0.8	1.24 (0.97, 1.60)	0.081	1.67 (0.80, 3.50)	0.2	1.07 (0.63, 1.81)	0.8	0.89 (0.05, 15.6)	> 0.9

*Note:* The ORs are all referenced to the normal sleep duration group. Boldface indicates statistical significance. The models were all adjusted for age, gender, race, education level, marital status, poverty status, smoking, alcohol consumption, body mass index, diabetes, hypertension, coronary heart disease, and angina.

Abbreviations: AMD, age‐related macular degeneration; CI, confidence interval; DR, diabetic retinopathy; OR, odds ratio.

^a^Among participants with diabetes mellitus, additionally adjusted for diabetes duration and HbA1c.

## 4. Discussion

Sleep plays a critical role in maintaining homeostasis, and sleep deprivation represents a significant stressor that impacts the brain and multiple physiological systems, potentially including ocular health. By analyzing a nationally representative sample of 5231 individuals aged 40 years and older in the United States, we found that, compared to normal sleep duration, the prevalence of visual impairment and major eye diseases was higher among those with long sleep duration. After adjusting for multiple confounders, long sleep duration was significantly associated with any objectively determined ocular disease, visual impairment, and glaucoma. Further stratified analysis by diabetes and hypertension status revealed that, in the nondiabetic population, long sleep duration was significantly associated with glaucoma, any objectively determined ocular disease, and visual impairment. In the hypertensive population, a significant association was observed between long sleep duration and glaucoma. These findings suggest that long sleep duration may be a potential risk factor for visual impairment and major eye diseases, although this is a cross‐sectional study and a causal relationship cannot be established at this time.

Ramos et al. [22] found a significant association between visual impairment and both short and long sleep duration in the US population using the 2009 National Health Interview Survey. A previous study in a Chinese population, which assessed self‐reported visual impairment by asking individuals about poor distance and near vision, found a prevalence of 34.2%. This study also observed that the prevalence of both short and long sleep duration was significantly higher in the visually impaired population compared to the normal‐sleeping population [23]. These studies are partially consistent with the findings of the present study, which show that long sleep duration is associated with visual impairment. However, unlike these studies, the present study did not find a clear association between short sleep duration and visual impairment. This may be related to differences in the study populations and the types of adjustments for confounders. In the present study, we considered more potential confounders that could have influenced the results. As this was a cross‐sectional study, a causal relationship could not be established. Further prospective studies are needed to verify this link.

A large national cross‐sectional study in Korea found that the overall prevalence of open‐angle glaucoma in the population was 3.91%, and that the prevalence of open‐angle glaucoma was higher in individuals with short sleep duration compared to those with long sleep duration [11]. This trend was even more pronounced in the obese population. The authors suggest that sleep disorders may contribute to glaucoma, particularly in obese populations. Data from a large prospective cohort study based on UK Biobank data also found that both short and long sleep durations were associated with glaucoma, with similar results across different types of glaucoma [12]. Qiu et al. [13] found that the prevalence of glaucoma in the US population was three times higher in those who slept 10 h or more compared to those who slept less than 7 h. A study evaluating subjective and objective sleep quality and activity in glaucoma patients found that these patients spent more time in bed and stayed asleep longer, but had less efficient sleep compared to healthy individuals [24]. The association between sleep disorders and glaucoma may involve both mechanical and vascular factors [25]. Longer sleep duration and poorer sleep quality may both be risk factors for and consequences of glaucoma.

With respect to DR, Jee et al., in the 2008–2012 Korean National Health and Nutrition Examination Survey, reported significant differences in DR prevalence based on sleep duration [6]. They found that individuals sleeping ≤ 6 h or ≥9 h had a higher prevalence of DR compared to those sleeping 6–8 h. Analyses from the Singapore Malay Eye Study and the Singapore Indian Eye Study also showed that long sleep duration was associated with vision‐threatening DR [7]. Another study from the Singapore Eye Clinic identified an association between short sleep duration and moderate DR [26]. However, in the present study, we observed an association between short sleep duration and any retinopathy only in the unadjusted model and in the model adjusted for basic demographic characteristics. No significant association was found between short or long sleep duration and DR after adjusting for confounders.

Regarding cataracts, a longitudinal study in South Africa found that short sleepers were more likely to develop cataracts compared to normal sleepers, although this analysis did not adjust for potential confounding factors [8]. Additionally, previous studies have reported that individuals sleeping less than 6 h/day were at a higher risk of developing cataracts than those sleeping more than 9 h/day, with prolonged sleep seemingly providing a protective effect against cataracts [9]. In the present study, an association between long sleep duration and cataract surgery was observed only in the unadjusted model. Further research is required to clarify the relationship between sleep duration and cataracts.

A cross‐sectional case‐control study in the Czech Republic showed that both short and long sleep durations were associated with an increased risk of developing AMD, but only in males; no significant association was found in females [27]. In a further study on AMD characteristics, Khurana et al. found that longer sleep duration was associated with geographic atrophy [28]. Additionally, short sleep duration was significantly associated with neovascular AMD [29]. Despite these findings, the present study aligns with previous systematic reviews and meta‐analyses, as no association was found between either long or short sleep duration and AMD [10]. The discrepancies in results may stem from variations in regional ethnicities and the influence of different confounding factors.

The strengths of this study are that it is a survey covering a diverse population of age, race, and socioeconomic status in the United States; the sample is representative; the ophthalmic questionnaire and examination assessments are standardized; and it contains a variety of demographic characteristics that may confound the analyses. Limitations of this study, however, are that it was a cross‐sectional study and could not assess causal associations between sleep duration and visual impairment or major eye diseases. Future prospective or longitudinal study designs are needed to validate the findings and explore causality. Second, the use of cataract surgery history as a proxy for cataract status may underestimate the true prevalence of cataract, since individuals with unoperated or undiagnosed cataracts could not be identified. In addition, participants did not undergo comprehensive anterior segment examinations, so conditions such as early‐stage cataracts or glaucoma‐related anterior segment changes may have been missed. Third, nighttime sleep duration was self‐reported and only captured hours slept at night on weekdays or workdays. Information on sleep continuity, quality, and daytime naps was not collected in this study, which may have resulted in misclassification of sleep duration and limited the accuracy of sleep assessment. Fourth, unmeasured factors such as genetic predispositions, diet, or environmental influences might contribute to residual confounding in the observed associations, although we adjusted for several key factors that could potentially affect the results in this study.

## 5. Conclusion

In conclusion, this study examined the relationship between visual impairment and major eye diseases across different sleep durations using data from the NHANES database. Long sleep duration was significantly associated with visual impairment, glaucoma, and any objectively determined ocular disease. Public health strategies should prioritize raising awareness of the connection between sleep health and eye health. Further research is needed to explore the underlying mechanisms and confirm causal relationships, providing a foundation for targeted public health strategies.

## Conflicts of Interest

The authors declare no conflicts of interest.

## Author Contributions

J.Z. was responsible for the conception and design of the work, the acquisition, analysis, and interpretation of data and drafted the manuscript. D.Z. and X.Y. contributed to the acquisition and analysis of data and drafted the manuscript. T.X. contributed to the conception and design of the work, as well as the review and editing of the manuscript.

## Funding

No funding was received for this manuscript.

## Supporting information


**Supporting Information** Additional supporting information can be found online in the Supporting Information section. Table S1: Definitions of some confounding variables.

## Data Availability

The datasets used in this study are available from the NHANES repository at https://www.cdc.gov/nhanes/.
