# Supplementary material for: Exploring the Link Between Sleep Duration and Visual Impairment and Major Eye Diseases: National Health and Nutrition Examination Survey 2005–2008
Source: Biomed Res Int. 2025 Dec 28;2025:1229050. doi: 10.1155/bmri/1229050 (PMC12744952; doi:10.1155/bmri/1229050)
Supplement: Supplementary file 1 — Supporting Information Additional supporting information can be found online in the Supporting Information section. Table S1: Definitions of some confounding variables. [file BMRI-2025-1229050-s001.docx]

Supplementary Material

**Table S1.** Definitions of some confounding variables

| Poverty Status | Defined as Income at or above poverty if poverty income ratio ≥ 1; otherwise categorized as other. |
| --- | --- |
| Hypertension | Hypertension was defined as meeting any of the following criteria: systolic blood pressure ≥ 130 mmHg; diastolic blood pressure ≥ 80 mmHg; a response of "Ever told you had high blood pressure" as “Yes”; or a response of "(Are you/Is SP) now taking prescribed medicine" as “Yes.” |
| Coronary Heart Disease | Coronary heart disease was defined as a “Yes” response to the question, "Has a doctor or other health professional ever told {you/SP} that {you/s/he} . . .had coronary heart disease?" |
| Angina | Angina was defined as a “Yes” response to the question, "Has a doctor or other health professional ever told {you/SP} that {you/s/he} . . .had angina, also called angina pectoris?" |
